# Genistein Increases Epidermal Growth Factor Receptor Signaling and Promotes Tumor Progression in Advanced Human Prostate Cancer

**DOI:** 10.1371/journal.pone.0020034

**Published:** 2011-05-16

**Authors:** Hisae Nakamura, Yuwei Wang, Takeshi Kurita, Hans Adomat, Gerald R. Cunha, Yuzhuo Wang

**Affiliations:** 1 Experimental Therapeutics, British Columbia Cancer Agency, Vancouver, British Columbia, Canada; 2 The Vancouver Prostate Centre, Vancouver, British Columbia, Canada; 3 Department of Urologic Sciences, University of British Columbia, Vancouver, British Columbia, Canada; 4 Department of Obstetrics and Gynecology, Feinberg School of Medicine, Northwestern University, Chicago, Illinois, United States of America; 5 Department of Anatomy and Urology, University of California San Francisco, San Francisco, California, United States of America; The Hong Kong University of Science and Technology, Hong Kong

## Abstract

Genistein is an isoflavone found in soy, and its chemo-preventive and -therapeutic effects have been well established from *in vitro* studies. Recently, however, its therapeutic actions *in vivo* have been questioned due to contradictory reports from animal studies, which rely on rodent models or implantation of cell lines into animals. To clarify *in vivo* effects of genistein in advanced prostate cancer patients, we developed a patient-derived prostate cancer xenograft model, in which a clinical prostatectomy sample was grafted into immune deficient mice. Our results showed an increased lymph node (LN) and secondary organ metastases in genistein-treated mice compared to untreated controls. Interestingly, invasive malignant cells aggregated to form islands/micrometastasis only in the secondary organs of the genistein-treated groups, not in the untreated control group. To understand the underlying mechanism for metastatic progression, we examined cell proliferation and apoptosis on paraffin-sections. Immunohistological data show that tumors of genistein-treated groups have more proliferating and fewer apoptotic cancer cells than those of the untreated group. Our immunoblotting data suggest that increased proliferation and metastasis are linked to enhanced activities of tyrosine kinases, EGFR and its downstream Src, in genistein-treated groups. Despite the chemopreventive effects proposed by earlier *in vitro* studies, the cancer promoting effect of genistein observed here suggests the need for careful selection of patients and safer planning of clinical trials.

## Introduction

Prostate cancer (PCa) is the most commonly diagnosed noncutaneous malignancy among North American men [1]. In 2009, about 192, 280 new cases of prostate cancer were diagnosed, and 27, 360 deaths were reported in the United States, ranking it second highest in mortality after lung cancer [2]. Compared to the USA, the incidence and mortality rates of prostate cancer are considerably lower in Asia [3], [4], [5]. Immigration studies have shown that Asians, who have adopted the western diet after immigration to the USA, had a significantly higher prostate cancer incidence than natives in Asian countries [6], [7]. Among many dietary differences between North America and Asia, the difference in soy consumption is exceptional. It is estimated that Asians consume 20–50 times more soy-based foods per capita compared to North Americans [8]. Previous epidemiological reports have indicated an inverse correlation between soy consumption and PCa risk, suggesting soy's chemopreventive effects [9]. The primary active component of soy is an isoflavone called genistein (4,5,7-trihydroxyisoflavone) [10], [11], [12], [13]. Due to similar molecular structure to estradiol, genistein is known to exhibit weak estrogenic activity by binding to the estrogen receptor (ER) and thus modulating estrogen-regulated gene transcription in target organs [14], [15].

The anticancer effects of genistein have been well studied *in vitro* and reported in several cancer cell lines including leukemia, lung, prostate and breast [16], [17], [18], [19]. Data from earlier studies demonstrate that genistein has a wide spectrum of biological effects, which include: induction of cell differentiation and apoptosis [17], [18], [19], [20], inhibition of cell growth [19], [21], [22], [23], [24], and abrogation of signal transduction pathways [25], [26], [27]. Genistein, thus, has attracted a considerable amount of attention in cancer research especially for its inhibitory action on protein tyrosine kinase (PTK) activities that are important in cell survival and proliferation [25], [26], [27].

Despite such promising *in vitro* anticancer data, recent *in vivo* studies have reported contradictory results regarding genistein's effects on metastasis. Studies using TRAMP and PC-3 animal models have shown that genistein unexpectedly increased metastasis [28], [29], while another study utilizing a PC-3M *in vivo* model showed the opposite effect [30]. These reports in combination with inconclusive preliminary results from phase II clinical trials [31], [32] suggest a need for closer examination of the effects of genistein *in vivo* using models that are more clinically relevant than other conventional *in vivo* models that rely on usage of cell lines.

In our study, we have utilized a subrenal xenograft technique employing a low passage of patient-derived PCa specimen in non-obese diabetic, severe combined immune-deficient (NOD-SCID) mice. The human cancer xenografts faithfully preserve the histopathological and genotypical characteristics of the original clinical sample [33], [34]. Using our patient-derived PCa xenograft system, we have found that genistein at pharmacological dosage increases cell proliferation, decreases apoptosis and promotes metastasis in an advanced human PCa. Such tumor-promoting effects of genistein are associated with increased phosphorylation of EGFR and Src tyrosine kinases.

## Methods

### 1. Materials and animals

The LTL163a tumor line (www.livingtumorlab.com) is derived from a high-grade prostate cancer sample obtained from a patient, and the xenograft tumor transplant line was developed from this clinical tissue sample as previously described [33], [34]. Written consent was obtained from the patient as well as ethics approval from University of British Columbia-British Columbia Cancer Agency Research Ethics Board (UBC BCCA REB), Vancouver, Canada. Animal care and experiments were carried out in accordance with the guidelines of the Canadian Council of Animal Care (CCAC), and the use of animals for our experiments was examined and approved by the Animal Care Committee of University of British Columbia (permit #: A10-0100).

Briefly, tumor tissue was cut uniformly into multiple small pieces, 1×3×3 mm, and grafted under the renal capsule of NOD-SCID male mice that were 6–8 weeks old [33], [34]. Two pieces of tumor per kidney were placed under renal capsule. Host mice were supplemented with testosterone pellets (10 mg/mouse), which were prepared with a PARR pellet press (PARR Instrument Co., Moline, Illinois) and implanted subcutaneously to promote cell growth. The grafts were grown in the renal site for 60∼90 days, harvested, and portions of the grafts were fixed for histopathological analysis. To develop a tumor transplant line, harvested tumor grafts exhibiting rapid growth were cut into small pieces and re-grafted onto the kidney of new hosts. After three transplant generations, swollen lymph nodes were noted, which were bisected and histologically examined for metastasis. Once metastasis was confirmed, fresh lymph node tissues containing metastatic deposits were re-transplanted onto the kidney to develop a metastatic tumor line and designated LTL163a.

### 2. Genistein treatment

In order to study the pharmacological effects of genistein in metastasis of human prostate cancer, we chose to use LTL163a metastatic tumor line, which were derived from the 10^th^ transplant generation. The tumors were cut into 1×3×3 mm pieces and grafted into eighteen NOD-SCID male mice with each kidney having one tumor graft (2 grafts/mouse). Testosterone pellets (10 mg) were implanted subcutaneously as described above in all animals at the time of grafting to maintain an adequate serum testosterone level because genistein-treatment has been shown to decrease serum testosterone levels via the hypothalamic/pituitary/gonadal axis [35]. Seven days after grafting, the animals were randomly divided into three groups; control (untreated), low-dose and high-dose of genistein (purity >99%, LC Laboratories, Woburn, MA). Mice in the low-dose group were given genistein dissolved in peanut oil by gavage at 2 mg/day (80 mg/kg body weight/day). Mice in the high-dose group were given 10 mg/day (400 mg/kg/day) of genistein. Control mice received 0.1 ml of oil-vehicle only. All groups were fed the same rodent diet (PicoLab Rodent Diet 20) and given autoclaved drinking water. Genistein treatment was started one week after grafting and continued for three weeks. The grafts were grown on the kidney for a total of 4 weeks. At the end of the 4^th^ week, blood samples and organs (kidney with tumor grafts, liver, lung, spleen and lymph nodes) were collected for analyses.

### 3. Measurement of serum genistein levels

LC-MS (liquid chromatography-mass spectrometry) was used to measure the serum level of genistein. First, frozen serum samples were thawed on ice, and 5 µl of 1 µg/ml luteolin in ethanol was added to 25 µl of serum as an internal standard (IS). Extraction was carried out by vortexing with 80 µl of acetonitrile and 5 µl of 0.2 M HCl. The extract was clarified by centrifugation for 5 minutes at 20,000× g, and the supernatant collected, diluted 1∶1 with distilled water, and centrifuged for another 5 minutes. The resultant supernatants were analyzed. An Acquity UPLC with a 2.1×100 mm BEH 1.7 µM C18 column coupled to a PDA detector in line with a Quattro Premier XE (Waters, Milford, MA) was used with water and acetonitrile containing 0.1% formic acid as mobile phases. A 25–100% acetonitrile gradient from 0.2–2.0 minutes was used, followed by re-equilibration to starting conditions for a total run time of 4.5 min. The MS was run at unit resolution with 3 kV capillary, 120°C and 350°C source and desolvation temperatures, 50 and 1000 L/hr cone and desolvation N_2_ gas flows and Ar collision gas set to 5.0e^−3^ mbar. M/z 287>89 and 287>153 were used to detect luteolin IS, and m/z 271>91 and 271>153 were used for genistein with cone voltage and collision energies optimized for each. OD data from 210–600 nm was collected with the PDA detector and OD260 was also used as an endpoint. A 5 point linear calibration curve from 1–800 ng/ml with 218 ng/ml IS was used for quantitation (R2>0.99; bias<10%). Genistein-glucoronide was presumed to be the most common form of conjugate, and the levels were estimated from OD260 data with the assumption that the extinction coefficient is similar to that of genistein.

### 4. Histopathology and immunohistochemistry

Harvested tumor grafts were cut in half at the thickest point of the tumor. One half was fixed in 10% neutral buffered formalin for histological/immunohistological (IHC) analyses. The other half was snap-frozen for protein analysis. The fixed tissue was processed to paraffin and embedded with the midline cut surface facing down in the paraffin block, so that sections began at the original cut midline surface. Preparation of paraffin-embedded tissues and IHC analyses were carried out as previously described [34]. The primary antibodies used in this study are Ki67 (Dako, Carpinteria, CA), caspase-3 (Cell Signaling Technology, Danvers, MA), ERα (Santa Cruz Biotechnology, Santa Cruz, CA), and ER**β** (Biogenex Laboratories, San Ramon, CA). All tissue sections were counter-stained with 5% (w/v) Harris hematoxylin and coverslipped with Permount (Fisher/Thermo Scientific, Waltham, MA).

### 5. Local Invasion and metastasis analyses

To examine metastatic incidence, lung, liver, renal, pancreatic, lumbar and thoracic lymph nodes were collected from each mouse. Each of these organs was fixed and processed as above for IHC analysis. Sections were stained with an anti-human specific mitochondria antibody (Millipore, Billerica, MA) and screened for presence of metastatic cells. Images of IHC-stained sections from each organ were captured using an AxioCam HR CCD mounted on an Axioplan 2 microscope and Axiovision 3.1 software (Carl Zeiss, Toronto, ON), with final magnifications of ×400. Percentages of animals containing human metastatic cells in either lumbar or thoracic lymph nodes were calculated. (In renal and pancreatic lymph nodes, all animals in three groups showed evidence of human cancer cells regardless of treatment thus omitted in the calculation.) For lung and liver, the number of positively stained cells was counted within randomly-selected microscopic fields per specimen.

### 6. Cell proliferation and apoptosis analyses

Proliferation index (PI) was assessed by IHC using a human specific proliferation marker, Ki67. Images of IHC-stained sections from each graft were captured as above. The mid section of the graft with the most densely populated human cancer cells was selected for imaging for all tumors. The numbers of Ki67 positive and negative-human cells were counted within the entire microscopic field for each graft of all animals and averaged. Based on the averaged cell count for each treatment group, PI was calculated as follows: PI (%) = (Ki67 positive cell count/total human cell count)×100.

Similarly, Apoptotic Index (AI) was calculated from cleaved-caspase-3 stained IHC sections of tumor grafts. The number of apoptotic cells per 10,000 human cells was counted for each tumor graft and averaged. AI was calculated as above.

### 7. Western blot Analysis

The remaining halves of the harvested tumor tissues were snap-frozen for protein analysis, from which mouse kidney tissues were surgically removed using a dissecting microscope prior to freezing. These frozen tissues were homogenized using a mortar and pestle in NP-40 lysis buffer (150 mM sodium chloride, 1.0% NP40, and 50 mM Tris-HCl pH 8.0) on ice, centrifuged at 12,000 rpm for 20 minutes at 4 degrees Celsius in a microcentrifuge, and supernatant was collected. The bicinchoninic acid (BCA) assay (Thermo Scientific, Waltham, MA) was performed according to the company's instructions to measure protein concentration for each tumor sample, and absorbance was measured at 562 nm. For gel electrophoresis, protein samples were boiled for 5 minutes, and 20 µg of protein was separated on SDS-PAGE (polyacrylamide gel electrophoresis) gel and electrotransferred to a PVDF membrane. The membrane was blocked with 5% bovine serum albumin (Sigma-Aldrich, St. Louis, MO) or 5% non fat milk in Tris Buffered Saline (pH 7.4) containing 0.1% Tween 20 (TBST) and incubated with primary antibodies to alpha-actin, phospho (p)-tyrosine (Santa Cruz Biotechnology, Santa Cruz, CA), total EGFR, p-EGFR (tyr1068), p-Src (tyr416) and total Src (Cell Signaling Technology, Danvers, MA). After primary antibody incubation, the membranes were washed with TBST and probed with appropriate HRP-conjugated secondary anti-mouse (Pierce, Rockford, IL) or anti-rabbit (Fisher/Thermo Scientific, Waltham, MA) antibodies. To detect proteins of interest, an enhanced chemiluminescence Western Blotting kit was used (Pierce, Rockford, IL).

### 8. Statistical Analysis

Metastatic Incidence, comparison of serum levels, PI and AI were evaluated using a two-tailed unpaired Student's *t*-test. Differences were considered statistically significant if *p* values were smaller than 0.05.

## Results

### Serum level of genistein

**Figure 1:**
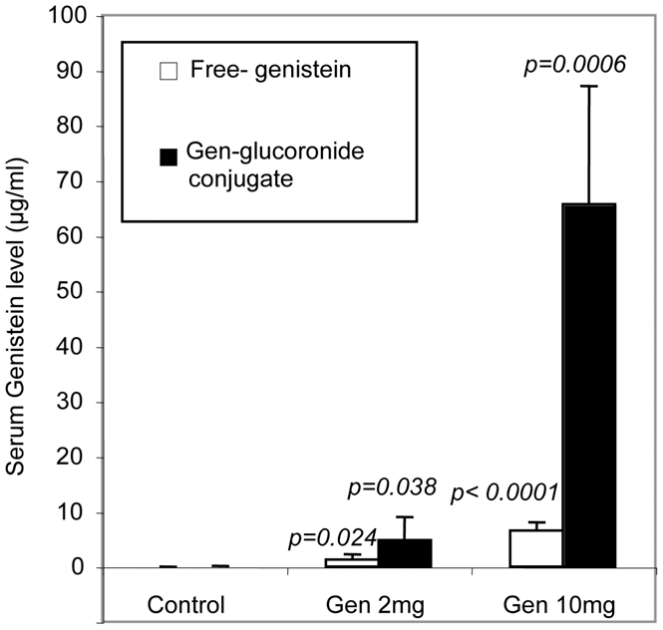
Serum levels of genistein measured by LC-MS. For treatment, six animals in low-dose group were given genistein dissolved in peanut oil by gavage at 2 mg/day (80 mg/kg body weight/day). Six mice in the high-dose group were given 10 mg/day (400 mg/kg/day) of genistein. Five control mice (one died before the end of treatment) received 0.1 ml of vehicle only. Blood was collected at the end of the 3-week treatment. *Columns*: mean serum concentration of genistein ± SD.

To ensure that genistein-treated groups received a pharmacological dosage of the compound, serum concentration of genistein was measured using high-performance liquid chromatography- mass spectrometry (HPLC-MS). Our HPLC-MS analysis showed that the untreated control group had a very low trace of serum genistein (0.002 µg/ml), whose most likely source is their chow (PicoLab Rodent Diet 20, Delta, BC). In comparison, low-dose and high-dose treated animals had 1.30 µg/ml to 6.63 µg/ml of free genistein, respectively. In our treated mice, ingested genistein formed glucuronide conjugates, and only a small portion of the genistein remained as free aglycones in the blood. The levels of both free and conjugated genistein were significantly higher in low (*p*<0.04) and high dose-treated mice (*p*<0.0006) than in the control group (Figure 1).

### Tumor growth of patient-derived PCa xenograft after genistein treatment

Effects of genistein on metastasis of advanced human PCa were examined using our patient-derived PCa xenograft tumor transplant line, LTL163a, which was developed from a prostatectomy sample of advanced stage and selected for metastatic activity. Tumors from the 10^th^ transplant generation of LTL163a tumor line were cut into uniformly sized pieces (1×3×3 mm) and grown under kidney capsules (one tumor piece/kidney, two tumor grafts/mouse) of eighteen male NOD SCID mice supplemented with a 10 mg subcutaneous testosterone pellet. Seven days after grafting, the host animals were randomly placed into three treatment groups; untreated-control, low-dose and high-dose genistein. Oral administration of genistein or oil (for control group) was continued for three weeks. At the time of harvest, enlarged kidneys with well-grown tumors were noted for all three groups. The average volume of tumors for control, low-dose and high-dose groups are; 650 mm^3^, 655 mm^3^ and 670 mm^3^, respectively. Despite increasing tumor volume trend for genistein treatment, there was no statistical significance among the groups. In addition to the upward expansive tumor growth from the kidney surface, tumors from all three groups invaded locally into the kidney (data not shown).

### Genistein promotes lymph node and secondary organ metastasis

**Figure 2:**
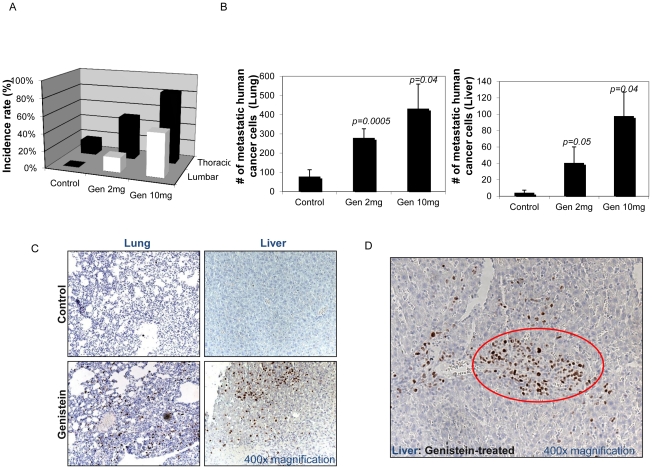
Genistein increases lymph node and secondary organ metastasis. At the time of harvest, lung, liver and four lymph nodes including renal, pancreatic, lumbar and thoracic nodes were collected from all animals. Each of these organs was fixed, processed, stained with an anti-human specific mitochondria antibody and screened for presence of human metastatic cells. Images of IHC-stained sections from each organ were captured using an AxioCam HR CCD mounted on an Axioplan 2 microscope with final magnifications of ×400. a) Percentages of animals containing human metastatic cells in either lumbar or thoracic lymph nodes were calculated. In renal and pancreatic lymph nodes, all animals in all three groups showed evidence of human cancer cells regardless of treatment (data not shown) thus omitted in the calculation. However, genistein-treated groups showed increased incidence of metastasis in lumbar and thoracic nodes compared to the untreated control group. The metastatic spread into lumbar and thoracic lymph nodes are presented as mean percentage in mice carrying LTL163A tumor grafts from three groups (untreated-control, low-dose and high-dose genistein). *Black column: metastatic incidence of thoracic lymph node. White column: metastatic incidence of lumbar lymph node*. b) For lung and liver, the number of positively stained cells was counted within randomly-selected microscopic fields. *Column*: mean number of invading cancer cells per microscopic field observed in lung and liver of the three groups ± SD. Results were statistically analyzed by t-test at the 95% confidence interval. c) Ki67-IHC staining of representative sections of secondary organs (liver and lung) from untreated-control and genistein-treated mice. Ki67 antibody used in this study is human specific. All magnifications are ×400. d) Ki67-IHC staining of liver from a genistein-treated animal showing aggregation of invading cancer cells. Red circle indicates a small island/micrometastasis focus containing >75 human cancer cells, which was observed only in genistein-treated mice. Magnification is ×400.

In order to assess the distant spread of tumor cells, we collected pancreatic, thoracic, lumbar and renal lymph nodes as well as internal organs. All groups showed evidence of human cancer cells in renal and pancreatic lymph nodes regardless of treatment (data not illustrated). However, in lumbar and thoracic nodes, control group had no or low incidence of metastasis (0% in lumbar and 17% in thoracic). In contrast, both low- and high-dose genistein treatments showed higher metastatic incidence in these two nodes compared to the untreated control group (Figure 2a); (17% for low-dose and 50% for high-dose in lumbar node. 50% for low-dose and 83% for high-dose in thoracic node). Similarly, the numbers of metastatic cancer cells in secondary organs such as lung and liver were significantly higher in genistein-treated groups than control (Figure 2b). Although scattered cancer cells were observed in secondary organs of all mice (Figure 2c), only genistein-treated mice showed aggregation of cancer cells (>75 cell aggregates) in those organs to form islands/micrometastasis as shown in Figure 2d. Thus, by several criteria, genistein had a dose-dependent metastasis promoting effect on LN and secondary organs in our model.

### Genistein affects cell proliferation and apoptosis

**Figure 3:**
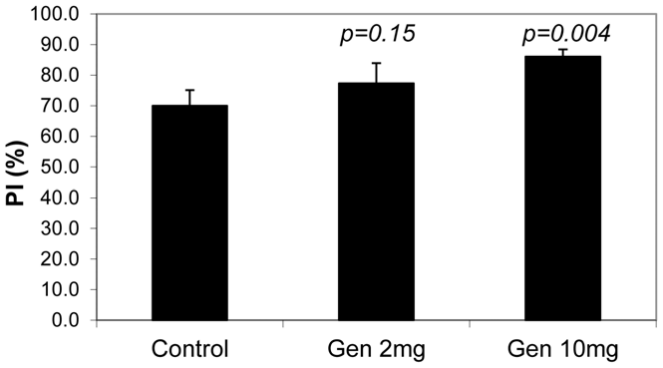
Genistein stimulates tumor cell proliferation. Proliferation index (PI) was assessed by IHC using a human specific proliferation marker, Ki67. The numbers of Ki67 positive and negative-human cells were counted within randomly selected microscopic field for each tumor graft of all animals and averaged. Proliferation Index (%): (Ki67 positive cell count/total human cell count)×100.

Because our data showed that genistein promoted spread of human cancer cells in the mouse hosts, we investigated its effects on tumor cell proliferation and apoptosis. Immunohistochemistry using a human-specific Ki67 antibody was performed to measure the rate of tumor cell proliferation. The result showed strong positive intensity within tumor grafts of all groups as well as in the locally invaded region of the mouse kidney (data not shown). Proliferation index (PI) was measured from the mid-section of tumor graft area. The comparison between groups revealed that both low- and high-dose groups had higher PIs (77.3% and 86.1%, respectively) than untreated control (70.0%), but only in the high-dose group was significantly different from the untreated control group (*p = 0.004*) as shown in Figure 3.

**Figure 4:**
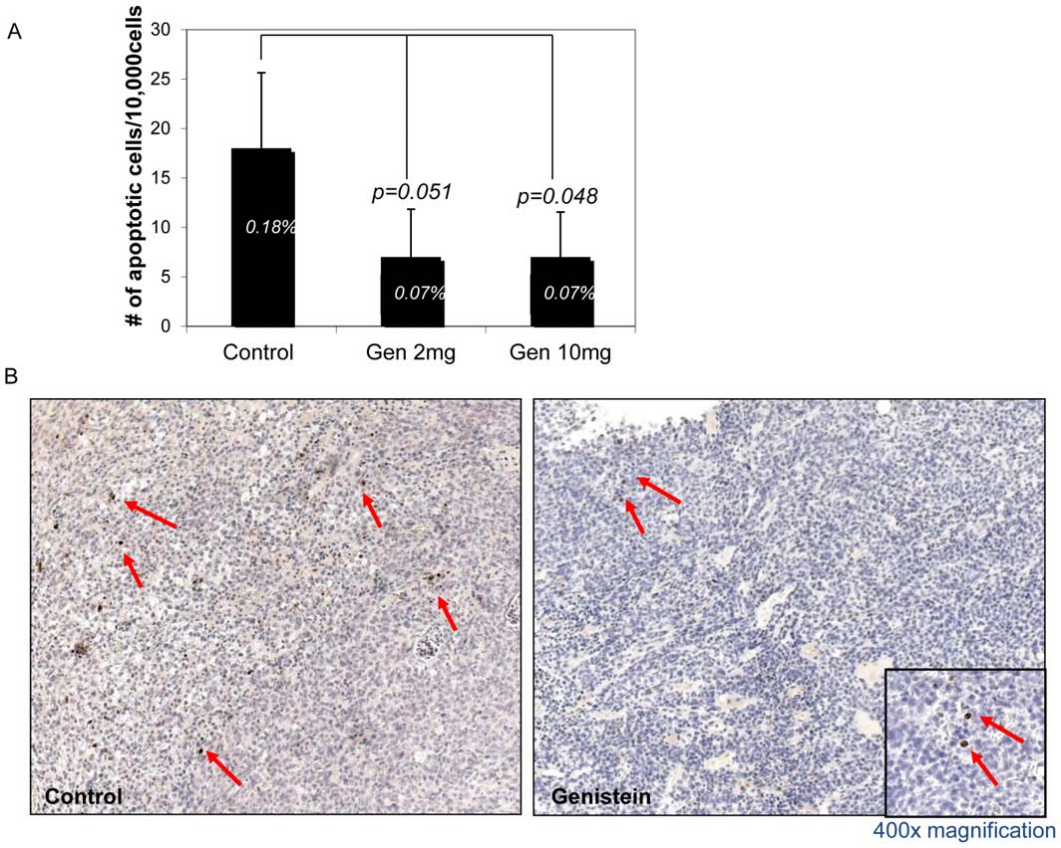
Genistein affects programmed cell death/apoptosis. Apoptotic Index (AI) was calculated from caspase-3 stained IHC sections of tumor grafts from all mice. The number of apoptotic cells per 10,000 human cells was counted for each tumor graft and averaged. a) Apoptotic Index (%) = (Caspase-3 positive cell count/total human cell count)×100. b) Caspase-3 immunohistological staining of tumor grafts from untreated and treated animals. Red arrows indicate positive/apoptotic cells. All magnifications are ×400. Inset is a magnified image of positive cells.

We also investigated whether genistein affected rates of programmed cell death, apoptosis. Immunohistochemical analysis with an anti-caspase 3 antibody revealed that tumors in genistein-treated mice had fewer caspase-3 positive cells than those in the control group (Figure 4). The control group had the highest apoptotic index (AI) of 0.18%, while both low- and high-dose groups had an AI of only 0.07% (*p = 0.05*: Figure 4a), indicating a significantly reduced rate of cell death in genistein-treated tumors.

### Genistein increases phosphorylation of protein tyrosine kinases

Previous *in vitro* studies have shown that genistein modulates tyrosine signaling pathways by altering kinase activities [25], [26], [27]. To determine if enhanced metastasis observed in the genistein-treated mice was linked to altered phosphorylation patterns, Western blot analysis was performed on tumor-derived protein lysates using an anti-phosphotyrosine antibody. Immunoblotting showed that genistein-treated tumors had higher level of phosphorylation at a 60 KDa protein band relative to the untreated control group. Interestingly, at longer exposures of the x-ray film, more intense band patterns were observed for higher molecular weight proteins in the genistein-treated group compared to control (Figure S1). Of particular interest was a band at 170 KDa. Longer exposure revealed bands in the genistein-treated group while no phosphorylation was observed in the control group at this particular sized protein.

**Figure 5:**
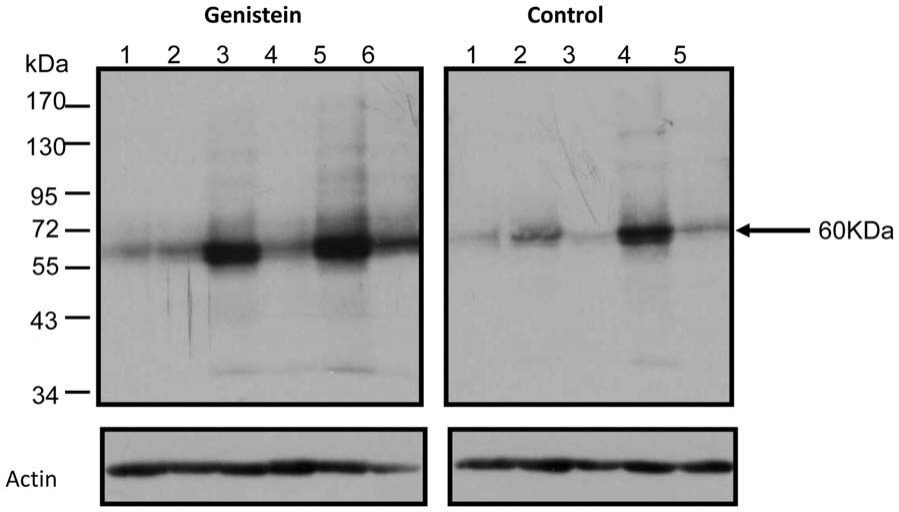
Effects of genistein on tyrosine phosphorylation. After three-week treatment of genistein, tumors were harvested, and protein was extracted from six genistein-treated and five untreated-control mice (one of the control mice died during treatment). Western blot analysis was performed on tumor-derived protein lysates for phosphotyrosine as described in Materials and Methods. The same blot was stained with an anti-alpha actin antibody to evaluate protein loading. Lanes represent proteins from individual tumors.

**Figure 6:**
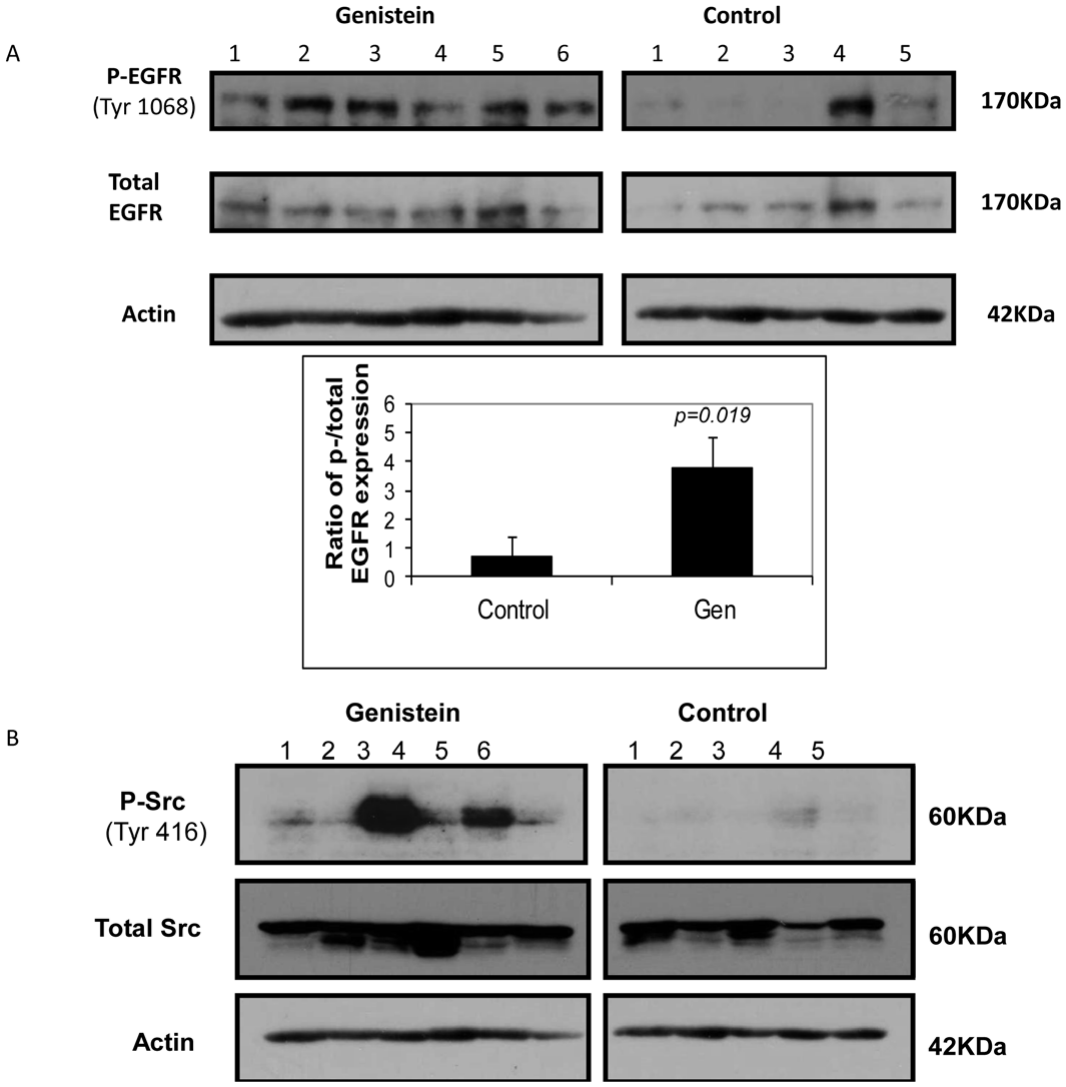
Effects of genistein on EGFR signaling. a) Genistein increases phosphorylation of EGFR at tyrosine 1068 residue. Below is the quantification of band intensity for phosphorylated EGFR from protein lysates of 5 untreated control and 6 genistein-treated tumors. *Columns*: mean ratio of phosphorylated EGFR/total EGFR protein band intensity ± SD. b) Genistein affects phosphorylation of Src at tyrosine 416 residue. Activated Src expression is significantly higher in genistein-treated tumors than that in untreated control. Lanes represent proteins from individual tumors.

To investigate further, we performed immunoblotting with antibodies targeting specific tyrosine kinases. The possible candidates for the 60 KDa and 170 Kda proteins (arrows in Figure 5 and Figure S1) are Src and its potential upstream molecule, epidermal growth factor receptor (EGFR). As shown in Figure 6a, genistein increased total EGFR protein expression slightly when compared to control. While almost all untreated control tumors (except for one) showed very little to no phosphorylation of this protein, most genistein-treated tumors showed high band-intensity (Figure 6a). Similarly, Src, a target of EGFR, showed slight upregulation of total protein expression compared to the control group. Again, no phosphorylation of Src was observed in the control group (except one tumor at a longer exposure), while a significantly higher level of phosphorylation was observed in the genistein-treated tumor samples (Figure 6b). These specific EGFR and Src immunoblot band patterns (Figure 6) confirm the general p-tyrosine staining patterns observed earlier (Figure 5).

## Discussion

Prostate cancer is the second leading cause of cancer-related deaths in North America. Since the introduction of androgen ablation therapy by Huggins and Hodges in the 1940's, hormone manipulation has been used as the main therapy for advanced PCa [36]. Despite initial tumor regression following hormonal therapy, some remaining PCa cells may survive this therapy, acquire androgen independency and become hormone refractory [37]. Once the disease progresses to the hormone-refractory state, there is no effective treatment currently available [38]. Therefore, a considerable amount of effort has been invested into strategies to treat advanced PCa. Previous *in vitro* studies have suggested that genistein has chemotherapeutic potential on hormone-dependent cancer cell lines. However, genistein's *in vivo* actions have recently been challenged by contradictory reports [28], [29], [30], [39], [40], [41], [42]. For example, one study reported that genistein inhibited PC3 bone tumor growth in SCID mice [43], and another showed that genistein decreased lung metastasis in androgen receptor-negative PC3-M implanted mice, which were fed genistein-enriched chow [30]. In contrast, Raffoul *et al.* found that genistein ingestion lead to an increase in lymph node metastasis in their PC3 implanted animal model [29]. In 2009, Touny and Banerjee demonstrated biphasic effects of genistein using the TRAMP mouse (transgenic adenocarcinoma mouse prostate) model. Genistein inhibited poorly differentiated PCa in these mice when incorporated in their diet before tumor initiation (ie, when the mice were fed genistein-diet between 4∼12 weeks of age). However, if the genistein-diet was given to the older TRAMP mice at ages of 12∼20 weeks, when prostatic intraepithelial neoplasia (PIN) was already present, it promoted PCa progression and induced lymph node metastasis [28]. From such data, it can be implied that life-time moderate consumption of (or early exposure to) genistein is important in prevention of the disease, but that genistein may not exhibit chemotherapeutic effects *in vivo* once PCa has already been established or progressed to an advanced stage.

To resolve the controversy of genistein's effects *in vivo*, we developed a clinically relevant xenograft model that has been generated from a patient prostatectomy specimen. The resultant patient-derived prostate tumor line used in our study, LTL163a, has been passaged for only 10 generations in immune-deficient mice and has maintained the original histopathological and genotypical characteristics of the original clinical sample [33], [34]. Our data show that both low and high dose genistein treatments promote metastasis in this advanced human PCa transplant line. Although the tumor size was not significantly different between control and genistein-treated groups after 3 weeks of treatment, metastatic incidence was greater in genistein-treated vs untreated controls. The metastatic progression observed in our model was characterized by increased cell proliferation and decreased apoptosis. One of the possibilities for the non-significant difference in tumor size between groups while genistein treatment increased proliferation is that all tumor grafts regardless of treatment had reached the maximum outward growth within the renal capsule, after which point started to invade inwardly into the kidney, then to LN and to secondary organs.

It is intriguing to note that genistein exhibits biphasic effects in hormone-dependent cancer cell lines, depending on the dosage [28], [44], [45]. Studies with breast cancer cell lines have shown that low-dose genistein (0.1–25 µM) stimulated growth of estrogen-dependent cells, while high-dose genistein (50–100 µM) inhibited proliferation [8], [44], [45], [46]. Wang *et al.* also demonstrated a similar biphasic effect of genistein in a non-cancerous prostate epithelial cell line (RWPE cells) [47]. In our study, however, we did not observe biphasic effects of genistein with the dose range of 80 mg/kg/day and 400 mg/kg/day. In order for genistein to exhibit inhibitory effects on advanced PCa *in vivo*, it may require higher dose administration.

Genistein has been shown to modulate activities of PTKs [25], [26], [27]. PTKs play a central role in regulating cellular functions such as proliferation, apoptosis, differentiation and cell survival [48]. PTK activity is, thus, important in the process of carcinogenesis and metastasis. EGFR is a membrane-associated tyrosine kinase, and its activity is regulated by ligand-binding and by interactions with EGFR family member receptors [49]. Once this molecule is phosphorylated, the activated signal is passed down through signaling cascades, activating numerous downstream molecules, which ultimately affects cell division, proliferation and cell migration [49]. Abnormal signaling in EGFR-related pathways leads to uncontrolled cell growth and has been reported in many solid tumors such as breast, colorectal and head and neck and pancreatic cancers [49]. Src is a member of a non-receptor tyrosine kinase located in cytoplasm and is one of the downstream molecules of EGFR [50], [51]. Dysregulation of Src has been linked to oncogenesis process for many years [52], and it is also known to play a role in metastasis by modulating cell motility and invasive abilities in skin, breast and colon cancers [53]. Our data show that genistein increases phosphorylation levels of EGFR and Src in an advanced human PCa, which are linked to enhanced cell proliferation and decreased apoptosis. Although previous *in vitro* studies demonstrate that genistein inhibits some PTK activities and thus impedes cell growth in cultured cells, our study shows that it increases phosphorylation of EGFR and Src *in vivo*, which is in agreement with the results from a phase I clinical trial that reported a ‘surprising’ increase in protein tyrosine kinase phosphorylation after oral administration of genistein [54]. As demonstrated here, it is important to note the difference between studies utilizing cultured cells versus *in vivo* studies that more closely mimic clinical cancer [55]. What contributes to this difference may lie in the dynamic tissue interactions that exist in the tumor microenvironment unique to *in vivo* models. For example, growth factors released by the stroma or systemic hormonal influence on cells may affect their biology in ways that cannot be replicated by isolated cancer cells *in vitro*.

Because of genistein's preferential binding to ERβ [57], [58] and the high and exclusive expression of ERβ in our tumor line (no ERα expression; Figure S2), it can be hypothesized that genistein's tumor stimulatory effects observed in this study may be mediated via ERβ activation. It has been shown previously that non-genomic signaling of ERs can activate PTKs [47], [56]. In a non-tumorigenic prostate epithelial cell line (RWPE-1), which predominantly expresses ERβ, Wang *et al.* showed that genistein treatment at low concentrations (0–12.5 µmol/L) increased cell proliferation and activity of extracellular signal regulated kinase (ERK) 1/2. They have also shown that anti-estrogen treatment with ICI 182, 780 inhibited genistein-induced cell proliferation and activities of ERK1/2 [47]. Another study by Migliaccio *et al.* demonstrated that ligand-activated-ERβ/androgen receptor (AR) complex associated with Src, which then activated the Src/Raf-1/Erk-2 pathway, stimulating cell proliferation in LNCap cells [56]. As demonstrated by the LNCap and RWPE studies [47], [56], genistein-activated ERβ may stimulate the EGFR/Src signaling pathway, which leads to increased proliferation, reduced apoptosis and tumor progression in our model. More studies are needed to determine how estrogenic activities of genistein affect tumor growth *in vivo*.

In summary, this study has demonstrated that genistein promotes metastatic activity in advanced PCa. It is possible that genistein has heterogeneous actions through which promotes cancer growth and progression in certain subtypes of cancers while inhibiting other tumors due to differential ERβ expressions among patients. Some PCa cells may have higher expression of ERβ than other cancer cells, favoring the progression of the disease. Future studies focusing on genistein's estrogenicity and its interaction with ERβ will help identify the subtypes of cancers whose growth is promoted or suppressed by genistein, facilitating rational planning of future clinical trials and minimizing the impact of genistein's adverse effects on cancer patients. A better understanding of genistein's cancer promoting actions may lead to the discovery of new therapeutic avenues.

## Supporting Information

Figure S1 Effects of genistein on tyrosine phosphorylation. Western blots were performed for phosphotyrosine: same blot as Figure 5 except that the X ray film was exposed longer (30 seconds).
(TIF)

Figure S2 ERα-and ERβ-Immunohistological staining of tumor grafts from untreated-control and genistein-treated mice. All magnifications are ×400.
(TIF)
