# Supplementary figures and images for: Genistein Increases Epidermal Growth Factor Receptor Signaling and Promotes Tumor Progression in Advanced Human Prostate Cancer
Source: PLoS One. 2011 May 16;6(5):e20034. doi: 10.1371/journal.pone.0020034 (PMC3095647; doi:10.1371/journal.pone.0020034)

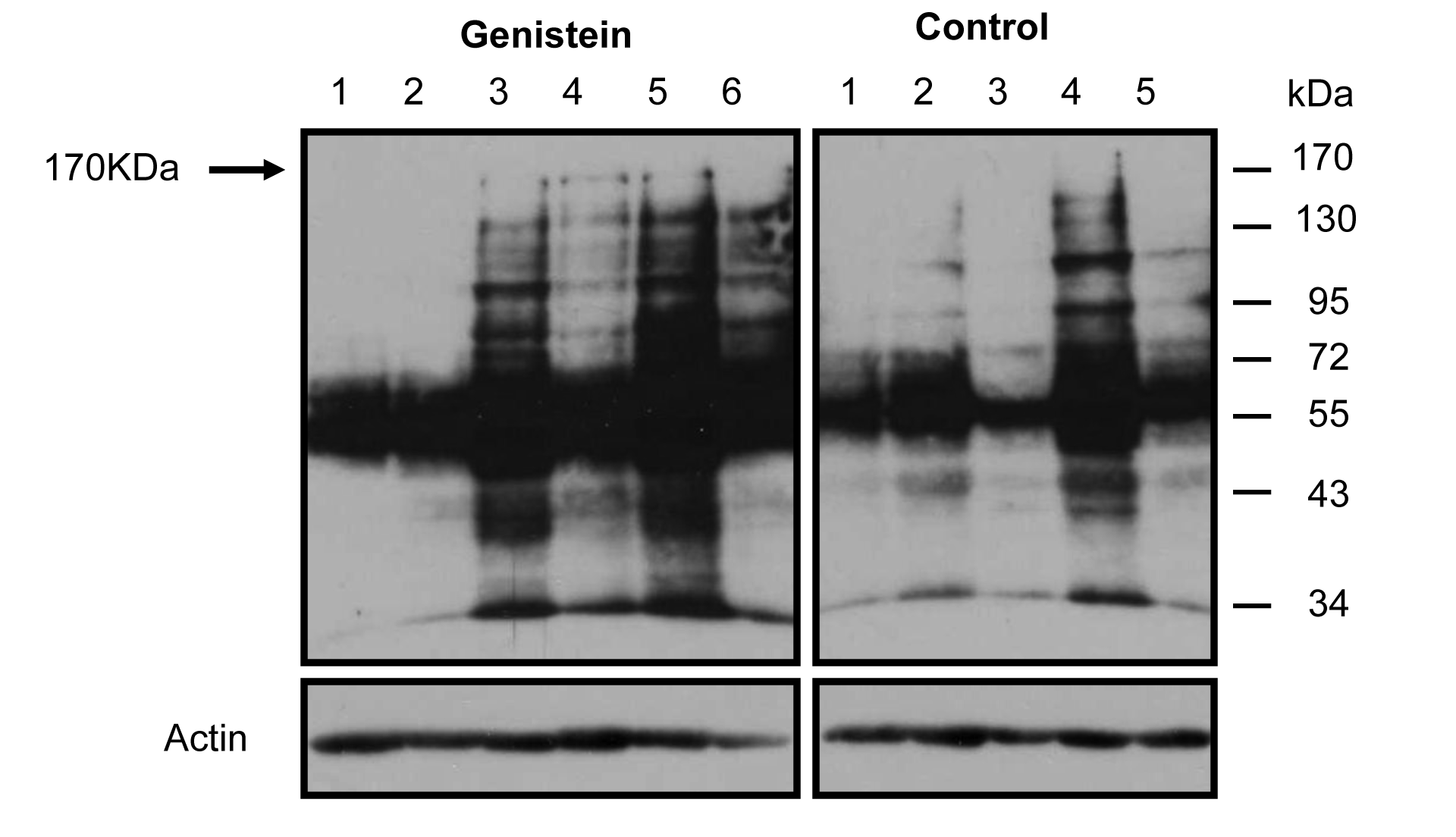

Supplement: Figure S1 — Effects of genistein on tyrosine phosphorylation. Western blots were performed for phosphotyrosine: same blot as Figure 5 except that the X ray film was exposed longer (30 seconds). (TIF) [file pone.0020034.s001.tif]

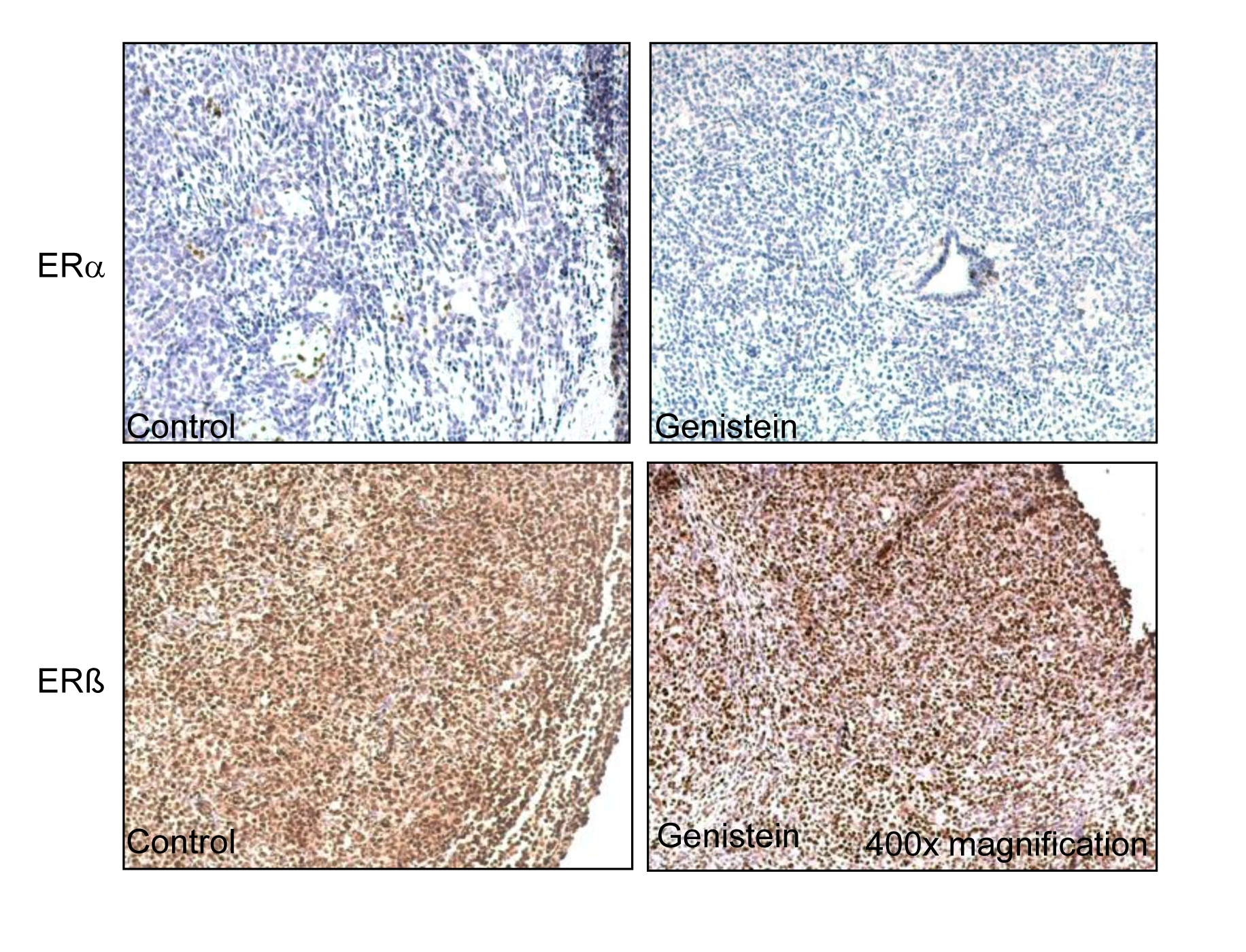

Supplement: Figure S2 — ERα-and ERβ-Immunohistological staining of tumor grafts from untreated-control and genistein-treated mice. All magnifications are ×400. (TIF) [file pone.0020034.s002.tif]
